# Protein Catabolites as Blood‐Based Biomarkers of Aging Physiology: Findings From the Dog Aging Project

**DOI:** 10.1111/acel.70226

**Published:** 2025-10-22

**Authors:** Benjamin R. Harrison, Maria Partida‐Aguilar, Abbey Marye, Danijel Djukovic, Mandy Kauffman, Matthew D. Dunbar, Blaise L. Mariner, Brianah M. McCoy, Yadid M. Algavi, Efrat Muller, Shiri Baum, Tal Bamberger, Dan Raftery, Kate E. Creevy, Dog Aging Project Consortium, Joshua M. Akey, Anne Avery, Brooke Benton, Marta G. Castelhano, Amanda E. Coleman, Kyle Crowder, Virginia R. Fajt, Annette L. Fitzpatrick, Unity Jeffery, Erica C. Jonlin, Matt Kaeberlein, Elinor K. Karlsson, Kathleen F. Kerr, Jonathan M. Levine, Paul Litwin, Jing Ma, Audrey Ruple, Stephen M. Schwartz, Sandi Shrager, M. Katherine Tolbert, Silvan R. Urfer, Benjamin S. Wilfond, Anne Avery, Elhanan Borenstein, Noah Snyder‐Mackler, Daniel E. L. Promislow

**Affiliations:** ^1^ Department of Laboratory Medicine and Pathology University of Washington Seattle Washington State USA; ^2^ University of Utah Department of Microbiology and Immunology Salt Lake City Utah USA; ^3^ Northwest Metabolomics Research Center, Department of Anesthesiology and Pain Medicine University of Washington Seattle Washington State USA; ^4^ Center for Studies in Demography and Ecology University of Washington Seattle Washington State USA; ^5^ School of Life Sciences Arizona State University Tempe Arizona USA; ^6^ Department of Clinical Microbiology and Immunology Tel Aviv University Tel Aviv Israel; ^7^ Blavatnik School of Computer Science Tel Aviv University Tel Aviv Israel; ^8^ Department of Small Animal Clinical Sciences Texas A&M University College Station Texas USA; ^9^ College of Veterinary Medicine and Biomedical Sciences Colorado State University Colorado USA; ^10^ Faculty of Medical & Health Sciences Tel Aviv University Tel Aviv Israel; ^11^ Jean Mayer USDA Human Nutrition Research Center on Aging Tufts University Boston Massachusetts USA

**Keywords:** aging physiology, amino acids, dogs, kidney, metabolome

## Abstract

Our understanding of aging has grown through the study of systems biology, including single‐cell analysis, proteomics and metabolomics. Studies in lab organisms in controlled environments, while powerful and complex, fall short of capturing the breadth of genetic and environmental variation in nature. Thus, there is now a major effort in geroscience to identify aging biomarkers that might be applied across the diversity of humans and other free‐living species. To meet this challenge, the Dog Aging Project (DAP) aims to identify cross‐sectional and longitudinal patterns of aging in complex systems, and how these are shaped by the diversity of genetic and environmental variation among companion dogs. Here we surveyed the plasma metabolome from the first year of sampling of the Precision Cohort of the DAP. By incorporating extensive metadata and whole genome sequencing, we overcome the limitations inherent in breed‐based estimates of genetic effects, and probe the physiological basis of the age‐related metabolome. We identified effects of age on approximately 36% of measured metabolites. We also discovered a novel biomarker of age in the post‐translationally modified amino acids (ptmAAs). The ptmAAs, which are generated by protein hydrolysis, covaried both with age and with other biomarkers of amino acid metabolism, and in a way that was robust to diet. Clinical measures of kidney function mediated about half of the age effect on ptmAA levels. This work identifies ptmAAs as robust indicators of age in dogs, and points to kidney function as a physiological mediator of age‐associated variation in the plasma metabolome.

## Graphical abstract

The Dog Aging Project identifies protein catabolites as biomarkers of age and aging physiology in the Precision Cohort of companion dogs across the United States.

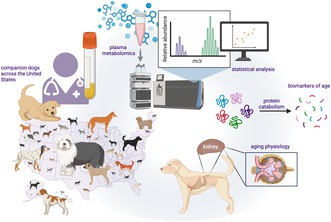

## 1 Introduction

Lab‐based studies on the biology of aging have led to major advances (Fontana et al. 2010; López‐Otín et al. 2023). However, it is not clear how lab discoveries apply to aging in the real world, where variation in genotype, environment, and their interaction all present major challenges to translational geroscience (Partridge et al. 2018). More recently, modern molecular tools have made it possible to identify biomarkers for age, morbidity, and mortality through the study of ‐omic domains, including the epigenome, transcriptome, metabolome, microbiome, and proteome (López‐Otín et al. 2023). Among the ‐omic domains, here we focused on the metabolome, the collection of small molecules that make up the structural and functional building blocks of cells. Targeted metabolome profiles are typically measures of one to several hundred features. The metabolome integrates variation in vast numbers of environmental and genetic factors, whose effects converge onto a relatively small number of metabolomic endophenotypes (Panyard et al. 2022). The metabolome may thus reflect important axes of metabolic and physiological variation that underlie traits as complex as aging in nature.

One goal of gerontological research is to understand the causes and consequences of aging in humans. There are now many systems‐level studies of age and aging in human populations (e.g., Hannum et al. 2013; Horvath 2013; Lehallier et al. 2019; Peters et al. 2015; Wilmanski et al. 2021). There are, however, a few limitations common to human studies. First, with few exceptions (e.g., Kuo et al. 2022; van den Berg et al. 2023; Zhang et al. 2024), studies of humans are carried out using cross‐sectional designs, which introduce selection and survivor biases among other challenges (Nelson et al. 2020). The long average lifespan of humans introduces yet another challenge. In studies of middle‐age or older humans, meaningful follow‐up periods to assess either mortality risk or longevity of biomarkers exceed 10 years and may require 20+ years to achieve statistical power sufficient to identify the majority of biomarkers that portend future risk (Wang et al. 2023). We need aging models in species with shorter lifespans that parallel the complexity of the human environment and genetic variation. Into this space leaps the companion dog.

The companion dog has much to teach us about healthy aging and its associations with genetics and the environment (Creevy et al. 2016). Dogs vary tremendously, not only in size, shape, and behavior, but also in their patterns of aging. Breed life expectancy can vary by a factor of more than two, from relatively short‐lived giant breed dogs like Leonbergers and Mastiffs, to longer‐lived small breeds, such as Pomeranians and Border Terriers (Yordy et al. 2020). Because dogs live with us, they experience the same local and regional environmental variation that we experience; they have a healthcare system as sophisticated as ours; and they have wide‐ranging genetic variation. Most of these features are almost completely absent for laboratory models of aging. Moreover, the short lifespans of companion dogs relative to those of humans give researchers a chance to see the impact their discoveries have on both dog and human health in their own lifetimes (Creevy et al. 2022).

In 2020, the Dog Aging Project (DAP) began enrolling tens of thousands of companion dogs in the United States in a long‐term longitudinal study of normative aging (Creevy et al. 2022). The goal of DAP is to characterize the range of aging patterns in dogs, to discover the genetic and environmental factors that shape this variation, and to identify the mechanisms by which they do so. By combining information from owner‐reported surveys, detailed demography, environmental data, ongoing studies of cognitive and behavioral traits, whole genome sequencing, clinical chemistry, and systems biology, the DAP aims to identify how these factors influence dog health and healthy aging (Creevy et al. 2022).

Through analysis of DAP data, we aim to shorten the timeframe for both biomarker discovery and analysis of normative aging, and to quicken the pace by which we test aging interventions in animals that mirror human genetic diversity and environmental complexity (Barnett et al. 2023; Creevy et al. 2022; Kaeberlein et al. 2016; Urfer et al. 2017). The few studies of age on the dog metabolome have revealed substantial differences between either the metabolome of cells cultured from young or old dogs (Brookes and Jimenez 2021), or the blood plasma from dogs of a range of ages (Puurunen et al. 2022). Puurunen et al. (2022) analyzed serum from over 2000 dogs and found age‐associated variation in lipids, fatty acids, and amino acids, amidst substantial variation due to diet, sex, and breed.

Here we analyzed a panel of 133 aqueous metabolites measured in plasma collected from the Precision Cohort, which consists of a subset of DAP dogs recruited specifically for deep molecular profiling (Prescott et al. 2025). The data analyzed here were from 784 Precision Cohort dogs, representing a diversity of ages during their first year of enrollment in the Cohort. We found that over a third of metabolites measured in dog plasma were associated with age, and we highlight two specific groups of age‐associated metabolites, including acylcarnitines and post‐translationally modified amino acids (ptmAA). These metabolites have repeatedly appeared in studies of human age (Panyard et al. 2022), and here we investigated the physiological basis of age association with ptmAAs in particular. The only known source of free ptmAAs is the breakdown of protein, and we found additional evidence for protein catabolism within the metabolome. We found that clinical measures of kidney function at least partially mediate the age associations of the ptmAAs. These results suggest that ptmAAs accumulate with age among dogs and may serve as a biomarker of aging physiology.

## 2 Results

### 2.1 Study Subject: The Dog Aging Project Precision Cohort

**FIGURE 1:**
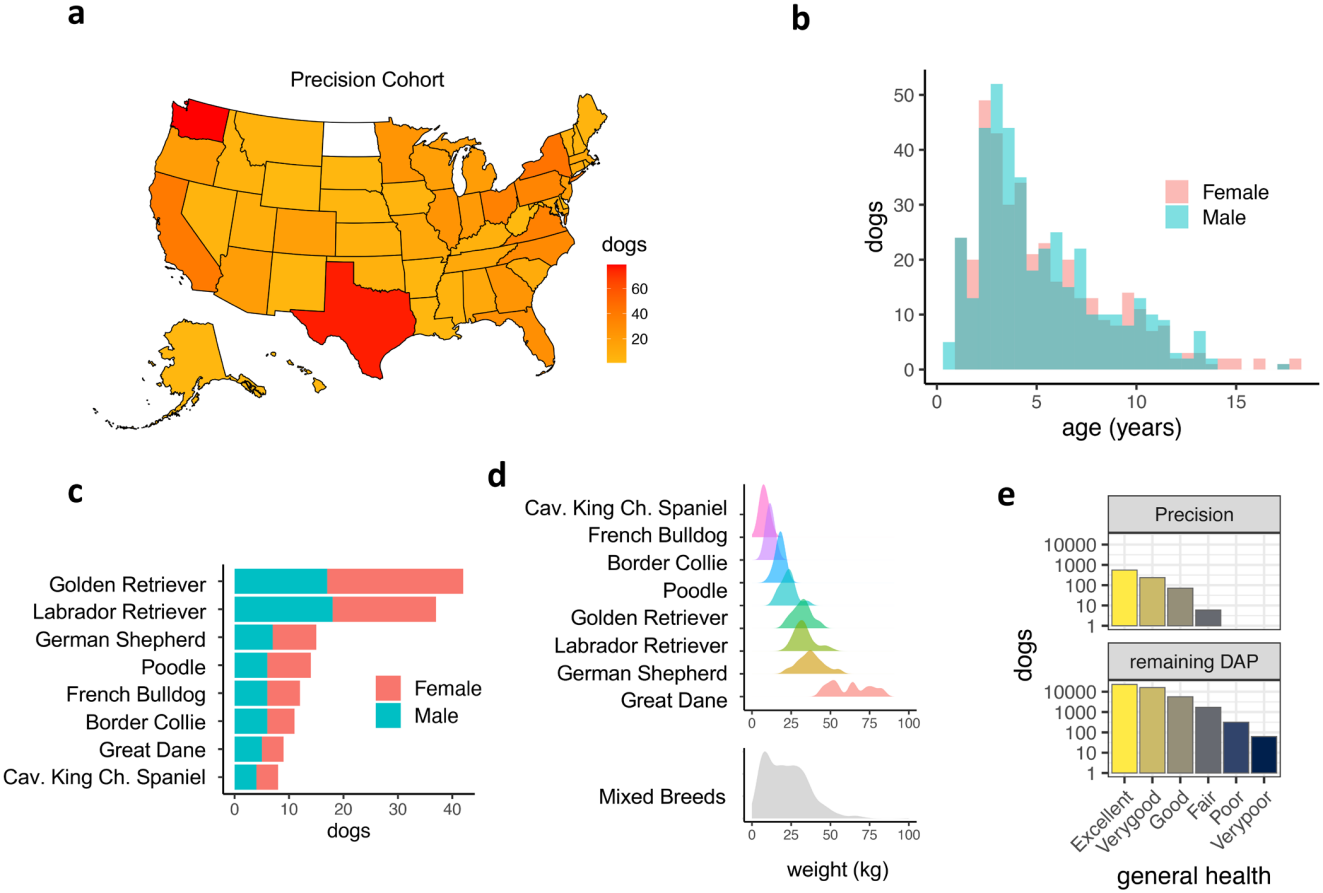
Demographic and health characteristics of the DAP Precision Cohort. (a) The geographic distribution of 784 dogs from the Precision Cohort. The number of dogs enrolled from each of the 50 United States is indicated by the color scale (range 1–79 dogs, white = 0 dogs). (b) The age distribution by sex for the Precision Cohort (c) Based on the ancestry estimated from among 115,427 SNPs (Methods), 148 of the dogs consist of one of eight common breeds (those with at least eight dogs and at least 85% ancestry), and that also have representation from both sexes. The remaining 636 dogs were of either an under‐represented breed, had ancestries from more than one breed, or did not include both sexes (Newfoundlands). (d) Dog weight at the time of blood collection for the most common breeds (upper panel), and for all other dogs (lower panel). (e) The number of dogs (note the log_10_ scale) at each general health category as given by owner reports. The upper panel summarizes the baseline Precision Cohort and the lower panel summarizes 47,444 dogs in the remainder of the DAP. Most Precision dogs (92%) were either in “Excellent” or “Very good” health, and no Precision dogs were categorized below “Fair”, whereas 0.8% of all DAP dogs were listed as being in “Poor” or “Very Poor” health. (f) Dogs reported to be in better general health were younger, on average, than dogs reported to be in worse health.

The Precision Cohort is a subcohort of the approximately 50,000 dogs that have so far been recruited into the Dog Aging Project. Beginning in January of 2021, 976 dogs were recruited into the Precision Cohort, representing a wide diversity of dog age, genetics, and geography. A summary of the cohort is shown in Figure 1. Here we analyzed metabolome data among 784 dogs from the first year of the Precision Cohort, which include 49% females and 51% males. Most dogs in the cohort were sterilized (83%). In designing the Precision Cohort within the overall DAP Pack, care was taken to recruit a cohort that reflects the full range of variation in American companion dogs in terms of geography, age, size, sex, sterilization status, and purebred versus mixed breed status (Creevy et al. 2022). The Precision Cohort dogs with metabolome data at baseline resided in all but 1 of the 50 United States (Figure 1a), with an average of 15.7 dogs per state and 21%, 60%, and 19% living in rural, suburban, and urban environments, respectively.

Among all dogs in the Precision Cohort, we identified evidence of ancestry from 110 different breeds (Sexton et al. 2025, Methods). The maximum proportion of ancestry assigned to a single breed in a given dog ranged from 4.0% to 99.9%, with 334 (43%) of the dogs having ancestry of 85% or more, our criterion for purebred designation (Morrill et al. 2022), and 450 (57%) mixed‐breed dogs. To analyze the influence of breed on the metabolome among breeds where we have the statistical power to do so, we chose purebred dogs for breeds in which there were at least eight dogs in the cohort, and with representation from both sexes. This resulted in 148 dogs from eight breeds (Figure 1c,d). Each of the remaining 636 dogs was among a less common purebred group or had mixed ancestries contributed by a median of 5 breeds per dog (range of 2–38), and these 636 were treated as a single group (“remaining dogs”) for this breed‐level analysis. Precision Cohort dogs were selected to represent the distribution of many traits within the overall DAP Pack, including general health. The Precision Cohort at baseline is generally healthy, with owner‐reported health, with 92% (*n* = 754) reported to have excellent or very good health status, and no dogs reported to have poor or very poor health, consistent with the low frequency of such dogs in the DAP (Figure 1e).

### 2.2 Targeted Plasma Metabolomics

**FIGURE 2:**
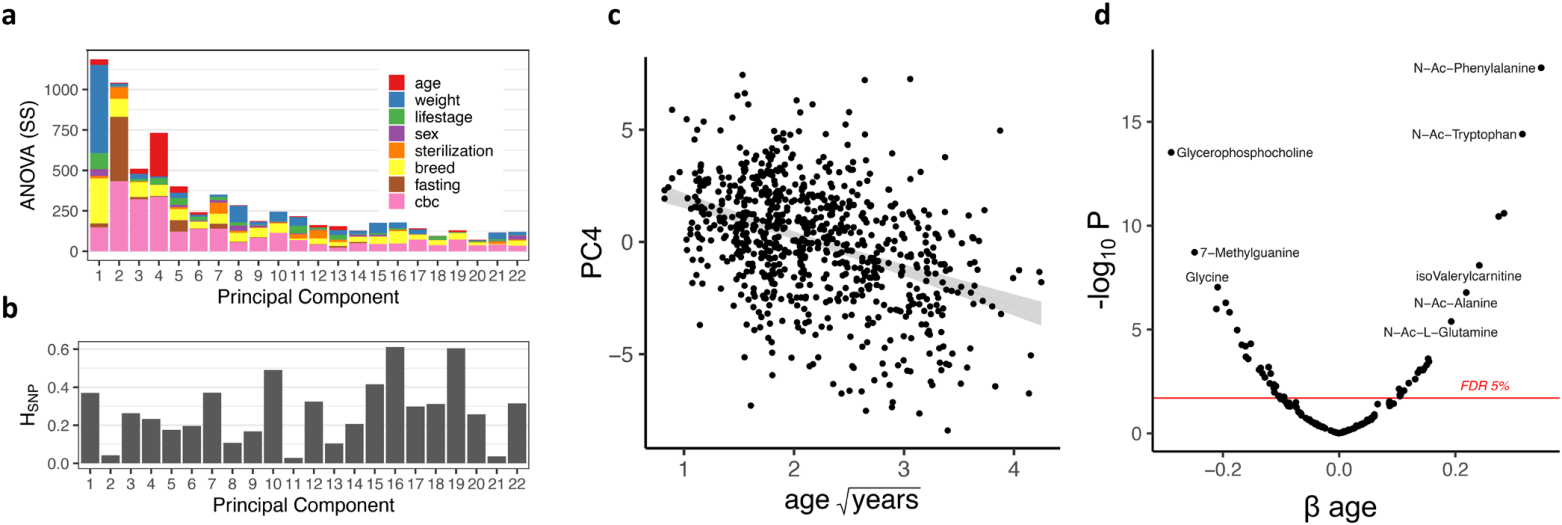
The age‐associated dog plasma metabolome (a) ANCOVA sum of squares (SS) among covariates (Methods) within the first 22 principal components (PC). The residual SS is not shown (see Figure S1). (b) The proportion of variation in each PC that was explained by the genetic relatedness among all dogs (H_SNP_, Methods). Note that the SS in (a) represents the total variance among the metabolome regardless of the PC, whereas in (b) H_SNP_ estimates the proportion of variance within each PC that was explained by relatedness. (c) PC4 associates strongly with age (square root‐transformed years), *β*_age_ = 1.69, *p* = 9.1 × 10^−13^, the shaded area is the 95% confidence interval for least squares regression. (d) Among the 133 plasma metabolites tested, the significance (−log_10_(P)) over the effect of age (*β*_age_) fit in a linear mixed model controlling for dog weight, sex, sterilization status, the duration of fasting prior to blood collection, CBC, and relatedness among the dogs (Methods). The FDR threshold of *α* = 0.05 is shown in red and representative metabolites are labeled.

The metabolome data consisted of 133 aqueous metabolites on a targeted liquid chromatography‐mass spectrometry (LC–MS) panel. The panel included amino acids and their derivatives, short‐chain fatty acids and fatty esters, nucleotides, carbohydrates, organic phosphates, and other metabolites. We first investigated shared variation among metabolites in dog plasma using Principal Component Analysis (PCA). By comparing the distribution of data across each PC to that expected from random data, we found evidence that each of the first 22 PCs captures non‐random variation (Tracy‐Widom test, *α* < 0.05); altogether, the first 22 PCs explain 60.3% of the variance in the metabolome (Figure 2a, Methods). Using Analysis of Covariance (ANCOVA), we estimated the variance of each PC that could be explained by age, weight, sex, sterilization status, life stage (puppy, young, mature or senior), breed, the duration of fasting prior to blood collection, and 17 complete blood count (CBC) variables (Methods). These variables together explained between 6.0% and 17.1% of the variance in each of the first 22 PCs (Figure 2a). Of all variables, weight explained the most variance of any PC at 7.0% for PC1, and age explained 5.8% of PC4 (Figure 2a). The CBC variables together explained up to 8.3% of the total variance among the first 22 PCs (Figure S1b), while common breed, life stage, sex, sterilization status, and the duration of fasting each explained less than 5.0% of any of the PCs. While accounting for less than 5% of the variance, breed effects on the metabolome manifest across 5 of the first 22 PCs (*p* ≤ 0.05, Figure 2a).

Given the difficulties associated with breed assignment in a genetically diverse cohort and the availability of low‐pass sequencing data, we chose to dissect the multivariate metabolome variation attributed to the eight common breeds using the available genetic data. Using linear mixed effects models, we measured the degree to which the effect of these breeds on the metabolome was affected when we simultaneously fit the genetic relatedness matrix (GRM, Methods). Adding relatedness to the model reduced the effect of breed by ~10% on average among the 5 PCs with breed effects, indicating that only a modest proportion of variation due to breed can be explained by genome‐wide relatedness (Figure S2). In contrast to the modest effects of breed, the proportion of variation in each PC that could be explained by the random effect of relatedness, the so‐called SNP‐heritability (H_SNP_, (Yang et al. 2011)), averaged 27%, ranging from 2.8% to 61.2% (Figure 2b). Therefore, in contrast to any genetic effects that may be represented by breed designation among the common breeds, the genomic data could hold more explanatory potential. In the following analysis, we used the GRM to control for the relatedness to ensure that genetic relatedness does not bias the results in our investigation of the effects of age on the metabolome. We included dog weight as a covariate to partially account for some of the additional breed‐level variation that could be explained by dog size (Figure 1d). Age, which ranged from 0.7 to 18.0 years among the Precision Cohort, explained a significant portion of the variance (*p* < 0.05) in 8 of the first 22 PCs, and was highly associated with PC4, where it accounted for 5.8% of the variance (Figure 2c, ANCOVA, *F*_1,751_ = 171.8, *p <* 2 × 10^−16^).

### 2.3 Effects of Age on the Dog Plasma Metabolome

The effect of age (*β*_age_) was statistically significant for 48 of 133 metabolites at a false discovery rate (FDR ≤ 0.05, Figure 2d, Table S1). Among age‐associated metabolites, we observed two enriched groups of metabolites—the carnitines and the free forms of ptmAAs. Four of the six carnitines on the targeted metabolome panel were age‐associated; three were more abundant in older dogs, and one, γ‐butyrobetaine (deoxycarnitine), was lower in older dogs. Of the 22 metabolites that increase with age, eight were either a carnitine derivative or a ptmAA (Figure 2d, Table S1).

### 2.4 Post‐Translationally Modified Amino Acids as a Biomarker of Age

**FIGURE 3:**
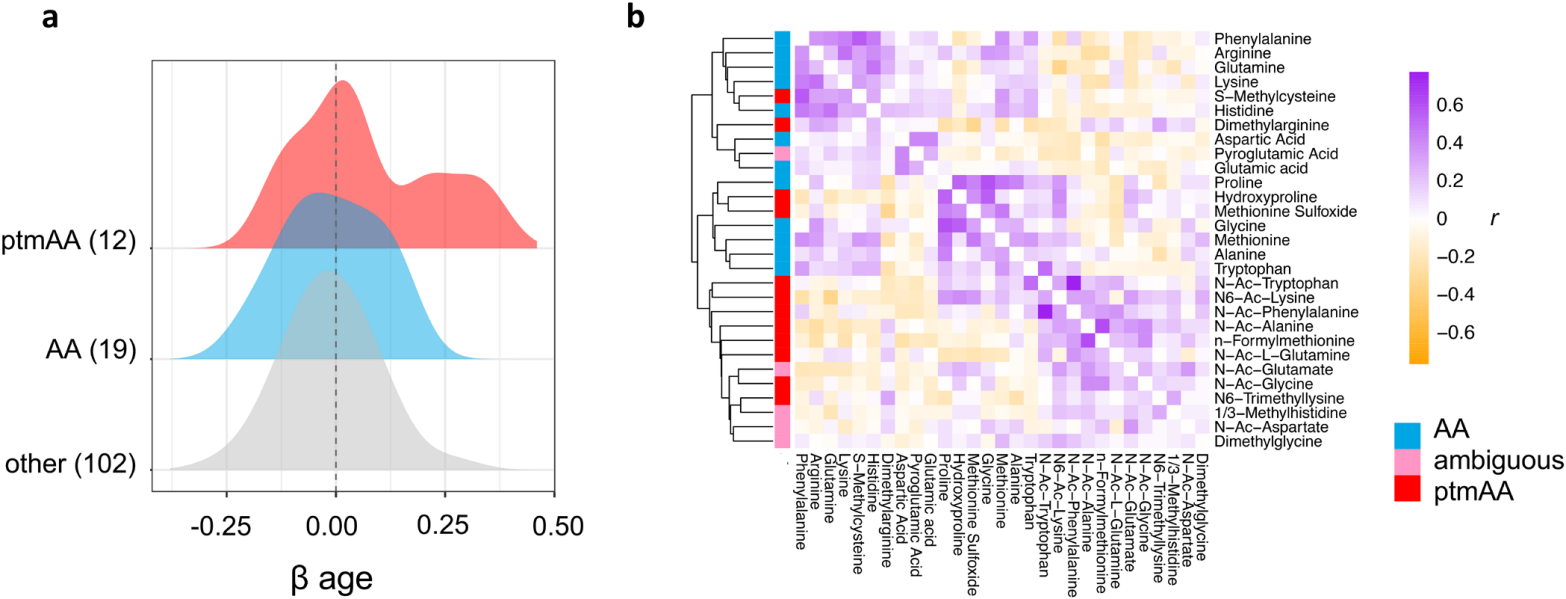
Post‐translationally modified amino acids associate with age and co‐vary in plasma. (a) The distribution of the effect of age (*β*_age_) fit to each metabolite in a linear mixed model controlling for covariates (Methods). The distribution of *β*_age_ among the 12 quantified post‐translationally modified amino acids (ptmAA, red), the 19 unmodified amino acids (AA, blue), and the 102 remaining metabolites of other classes (other, gray). (b) A heatmap of clustered pairwise correlation among the ptmAA, with the addition of five modified amino acids that are not necessarily post‐translationally modified and may form instead from de novo synthesis (ambiguous), the unmodified amino acids (parent AA). Values in the map are Pearson's *r* from correlations calculated among the residuals of the full mixed model, and so are adjusted for age, relatedness and the other covariates. The diagonal was made white for clarity. The rows are annotated by metabolite class and a dendrogram of UPGMA clustering (Methods) is shown at left.

Age affects 50% of the measured ptmAAs (Figure 3a, Table S1). Older dogs have a higher abundance of four ptmAAs: the N‐terminally acetylated (N‐Ac) phenylalanine, tryptophan, alanine, and glutamine. The two ptmAAs reduced in the plasma of older dogs compared to younger dogs were methionine sulfoxide and hydroxyproline. In this analysis, we distinguish a second kind of modified amino acid, the ambiguous type, that could be but are not necessarily post‐translationally modified, and may instead form by de novo synthesis. In this study, there were five such ambiguous modified amino acids: pyroglutamate, dimethylglycine, 1/3‐methylhistidine, N‐Ac‐aspartate, and N‐Ac‐glutamate, and we do not consider these metabolites to be ptmAAs given the ambiguity in their origins. The unmodified forms of each of the ptmAAs were also measured, and four of these unmodified amino acids were also age‐associated. We tested the hypothesis that the association between ptmAAs and age might be related to age‐association of the corresponding unmodified version of each of the ptmAAs. The *β*_age_ of ptmAAs was not correlated with the *β*_age_ of the unmodified amino acids (linear regression *r* ^*2*^ = 0.07, *p =* 0.25). Similarly, of the 12 ptmAAs, the six that were age‐associated did not correspond to which of the 12 corresponding unmodified amino acids were (Fisher's exact test, odds ratio = 0.70, *p* = 1).

There are two alternative explanations for the difference in abundance of ptmAAs in young and old dogs. First, there might be broad changes with age in the influx or removal of ptmAAs from the blood, from a common source, such as might be caused by differences in protein catabolism, or the removal of its byproducts. Alternatively, there might be a variety of processes that could generate or remove individual ptmAAs in plasma, such as the transport of specific ptmAAs from the gut to the bloodstream, or the degradation or excretion of select ptmAAs. While we do not rule out the latter possibility, we found empirical support for the former hypothesis. If protein catabolism broadly differs among young and old dogs, then, *a priori*, this should give rise to ptmAAs in rough stoichiometry to their abundance among digested proteins. Therefore, we would anticipate that, broadly, the abundance of ptmAAs would positively co‐vary in plasma. Alternatively, if ptmAAs were acquired, synthesized, or removed independently, without a common origin, their abundances would not be expected to correlate. We examined the correlation among metabolites after removing the effects of age and other covariates (Methods). By clustering the Pearson's correlation among both the ptmAAs, the ambiguous modified amino acids, and their unmodified forms, we found that each metabolite covaried positively with between 3 and 17 other metabolites (FDR < 0.05), and that the ptmAAs and unmodified amino acids tend to cluster separately (Figure 3b). The covariation among ptmAAs supports the hypothesis that there is a general age‐related shift toward protein catabolism and/or diminished removal of its byproducts in dogs.

### 2.5 Testing Putative Sources of Post‐Translationally Modified Amino Acids

The primary physiological sources of amino acids from protein catabolism are from proteolysis of dietary protein in the intestine, digestion of muscle and other tissue, and proteolysis in the liver (Denton and Elvehjem 1954; Levitt and Levitt 2018). The age association of ptmAAs could be caused by age‐related changes in metabolism, including the catabolism of either dietary protein or endogenous sources of protein, like the digestion of tissue. Alternatively, age may be accompanied by changes to the rates of clearance of ptmAAs from the blood. We used the available owner survey responses to evaluate variation in diet as a potential driver of ptmAA in plasma. Drawing on survey responses from 761 dog owners, we found these dogs vary substantially in their primary diet category from the most popular, dry kibble, to raw, canned, and freeze‐dried foods, including representation from both commercial and home‐prepared diets (Figure S3a). These primary diet categories were the most comprehensive data that we identified among the survey data. While limited to diet type, we tested each diet type for associations with plasma metabolites using dry kibble, the primary diet of 86% of P1 dogs, as the reference diet (Methods). There were up to 30 metabolites associated with at least one diet type, with the effects of home‐prepared (raw or cooked) and raw commercial diets being similar (Figure S3b). None of the age‐associated ptmAAs were associated with any of the primary diets. However, S‐methylcysteine, a ptmAA which was not age‐associated, was higher in dogs eating commercial raw and home‐cooked diets when compared to those eating kibble (Figure S3c).

As an additional means to identify effects of age while controlling for the effect of diet type, we tested for effects of age only among the 653 dogs that eat kibble as their primary dietary source. Even with reduced statistical power due to smaller sample size, all of the age‐associated ptmAAs remained age‐associated (FDR < 0.05). We lack more detailed data on diet composition and so did not further resolve dietary influences on the age‐associated metabolome. However, using the available data, we found no evidence that variation in primary food type among the dogs explains the abundance of ptmAAs in the plasma of older dogs.

### 2.6 Markers of Protein Catabolism and Kidney Function Covary With Post‐Translationally Modified Amino Acids

**FIGURE 4:**
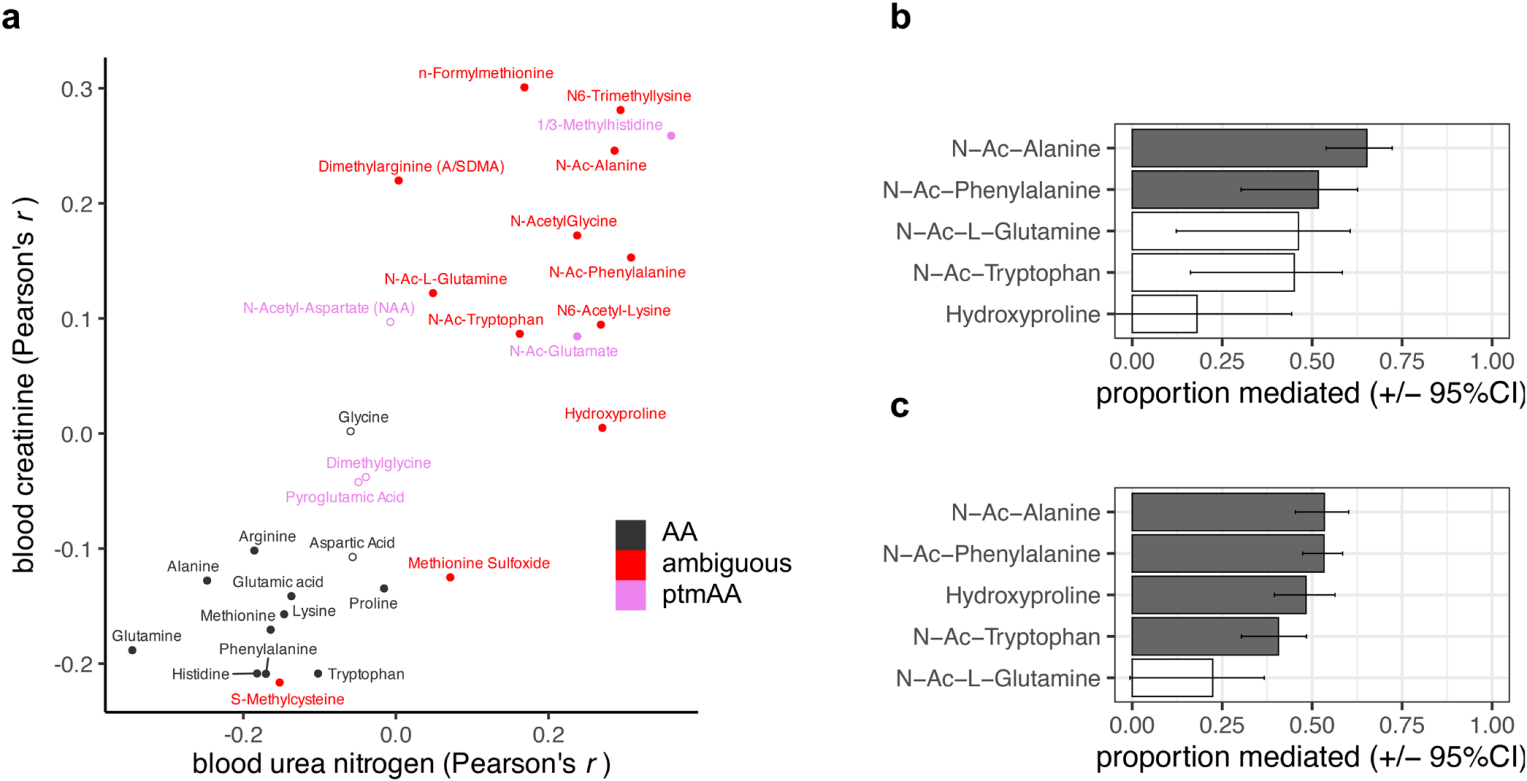
Indicators of protein metabolism and kidney function partially explain the age association of ptmAAs. (a) The Pearson's correlation between blood creatinine and each of the ptmAAs, the ambiguous ptmAAs, and unmodified parent amino acids (parent) is plotted over the Pearson's correlation of the same metabolites with blood urea nitrogen. (b, c) Of the five ptmAAs that associate with age and creatinine or BUN, the proportion of the age effect on ptmAAs in a full mixed model that is reduced (proportion mediated) by blood creatinine (b) or blood urea nitrogen (c). Error bars are 95% confidence intervals (Methods). Filled bars correspond to metabolites whose mediation effect is significant (FDR < 5%).

The kidneys play a key role in removing metabolomic byproducts to the urine, including the waste products of protein and amino acid catabolism (Schlosser et al. 2023). To test for effects of kidney function on plasma ptmAA, we evaluated several potential biomarkers. Serum creatinine and blood urea nitrogen (BUN) are common clinical metrics used to assess protein turnover and estimate the glomerular filtration rate (GFR) of the kidney (Dahlem et al. 2017; Hokamp and Nabity 2016; Yamaguchi et al. 2021). As expected by the protein catabolism hypothesis, the level of serum creatinine and of BUN correlate positively with the abundance of nine and eight of the 12 ptmAAs, respectively (mean *r* = 0.125 and 0.165, bonferroni *p* < 0.05), and among the unmodified AAs, BUN and creatinine were either not correlated or were negatively correlated (mean *r* = −0.101 and −0.109, bonferroni *p* > 0.05 Figure 4a). These ptmAAs, which include all the N‐acetylated amino acids, n‐formyl‐methionine, N6‐trimethyllysine, dimethylarginine, and hydroxyproline, all covary with creatinine and/or BUN (Figure 4a). Given that serum creatinine and BUN co‐vary with many of the ptmAAs in plasma, we tested the potential for either creatinine or BUN to act as a mediator of the elevated ptmAAs in the plasma of older dogs (Methods). By comparing the effect of age on each metabolite, with and without the addition of creatinine or BUN to a full mixed model, we found evidence that the age association of five of the six age‐associated ptmAAs was substantially mediated by either serum creatinine or BUN (Figure 4, FDR < 5%, Methods). Therefore, for the ptmAAs, if we account for serum creatinine, which is inversely proportional to the GFR, and BUN, a byproduct of protein catabolism, we can explain between 40% and 67% of the effect of age on the abundance of ptmAAs in plasma.

We sought alternative indicators of kidney function from clinical urinalysis of 741 of the Precision dogs. Among 10 urinalysis measures, both bilirubin and urine specific gravity (uSG) declined with age (FDR < 5%). We failed to find metabolites associated with bilirubin. However, there were 15 metabolites, including hydroxyproline, associated with uSG, and we find that 18% of the effect of age on hydroxyproline was mediated by uSG [proportion mediated = 0.18 (CI = 0.12–0.25), FDR < 5%]. No other ptmAA was associated with uSG. Urine specific gravity was not associated with serum creatinine (linear mixed model, *β*_creatinine_, *p* = 0.059), and therefore uSG could indicate kidney function that was independent of serum creatinine.

## 3 Discussion

We have surveyed the age‐related plasma metabolome among the diverse Precision Cohort of the Dog Aging Project. Amidst the genetic and environmental complexity of the companion dog, we found metabolites associated with CBC/Chem variables, dog weight, sterilization status, and the duration of fasting, as well as significant variation by breed, only some of which could be explained by finer‐scale genetic relatedness. In controlling for these covariates, we sought plasma biomarkers of age with higher translational potential. In doing so, we found substantial differences in the plasma metabolome of dogs by age, including ~36% of metabolites. The age‐associated metabolites were like those associated with age in humans (Darst et al. 2019; Houtkooper et al. 2011; Jarrell et al. 2020; Johnson et al. 2018; Lassen et al. 2023; Panyard et al. 2022; Robinson et al. 2020; Sol et al. 2023; Wang et al. 2023) and include byproducts of protein catabolism. We then query the available data for the Precision Cohort and identify diet and biomarkers of kidney function as potential mediators of parts of the age‐associated metabolome.

While most studies of dogs aimed at identifying biomarkers of age or health focus on dogs of a particular breed (Morelli et al. 2022; Qu et al. 2022) or on dogs living in the limited environment of a dog colony (Christie et al. 2009), the Precision Cohort of the DAP is similar to the large‐scale cohort of Puurunen et al. (2022), with the shared aim of a comprehensive representation of a wide range of dog genetic and environmental variation. Like Puurunen et al. (2022), we saw indications of genetic influences throughout the metabolome. Here we made use of genome‐wide allelic variation to show that a substantial portion of the metabolome was explained by fine‐scale relatedness among dogs, which points toward genetic influences on the metabolome that transcend breed level variation.

We acknowledge several likely sources for bias in this study cohort, all of which lead us to reason that the baseline characterization of age‐associations among the Precision Cohort may reflect healthy aging, rather than indicators of unhealthy aging (Nelson et al. 2020). A baseline cohort of any species, particularly one that recruits both younger and older participants, is inherently subject to survivor bias, where subjects in the study, particularly at later ages, can only represent the subset of individuals who have survived to that age (Anderson et al. 2011). Furthermore, an owner's decision to enroll a dog can be influenced by their perception of whether their dog is a good fit for the study, creating a self‐selection bias. For instance, owners may avoid enrolling dogs they perceive as “too young”, “too old”, or “too sick”. This mirrors the volunteer bias in human studies, where individuals with certain health status or demographics are often underrepresented (Fry et al. 2017; Galea and Tracy 2007). While acknowledging these caveats, we discuss our results as indications of age‐related variation in the plasma metabolome, and what it might indicate about the physiology of dogs as they age.

We used a mixed model framework to estimate age association in the plasma metabolome while simultaneously correcting for the covariates mentioned above. In doing so, we avoided the confounding influence of common variation among dogs with the aim of identifying age associations that are more likely to translate to dogs generally. We found a significant effect of age in 48 of 133 plasma metabolites (Figure 2; Table S1). We focus our discussion on two groups of metabolites—carnitines and the ptmAAs and other products of protein catabolism—for their similarity to age‐associated metabolites in humans, and regarding the ptmAAs, because they provide novel clues to the physiological differences between young and old dogs.

### 3.1 Age‐Associated Plasma Metabolites in Dogs, and Parallels in Humans

#### 3.1.1 Acylcarnitines

Among the most consistently age‐associated plasma metabolites in humans and mice are those involved in fatty acid metabolism, including the fatty acids themselves, as well as carnitine and acylcarnitines (Darst et al. 2019; Houtkooper et al. 2011; Jarrell et al. 2020; Lassen et al. 2023; Robinson et al. 2020; Sol et al. 2023; Wang et al. 2023). In this study, we limited our analysis to the aqueous metabolome, which lacks hydrophilic lipids. However, of the six carnitines measured, four associated strongly with age, with all but one, γ‐butyrobetaine, positively associated with age. Acylcarnitines and carnitine are required to shuttle fatty acids into mitochondria for β‐oxidation, and several authors speculate that the rise in acetylcarnitine with age in humans could be due to changes in fatty acid metabolism, or to reduced function of mitochondria (Jarrell et al. 2020; Lassen et al. 2023), or of the kidneys (Yamaguchi et al. 2021). γ‐butyrobetaine, the only carnitine negatively associated with age, is interconverted with carnitine, which was associated positively with age. The opposite relationship of carnitine and γ‐butyrobetaine therefore could be due to the age‐related change in balance between carnitine and γ‐butyrobetaine. Overall, the increased abundance of acylcarnitines in plasma among older dogs was consistent with patterns seen in humans, although the cause is unclear.

#### 3.1.2 Byproducts of Protein Catabolism

There are a variety of types of modified amino acids found in cells and tissue. However, as defined here, ptmAAs are only known to form on polypeptides (Ree et al. 2018). Therefore, the only known source of ptmAA, in the free forms measured by LC–MS, is protein catabolism. Studies of plasma metabolites often include measures of the amino acids, and in both dogs and humans, amino acid concentrations are regularly found associated with age (Darst et al. 2019; Houtkooper et al. 2011; Johnson et al. 2018; Panyard et al. 2022; Puurunen et al. 2022; Sol et al. 2023; Wang et al. 2023). Because amino acids can be synthesized de novo or generated from proteolysis, the physiological source of age‐associated amino acids has not been identified, though several studies speculate that protein catabolism may vary by age (Lawton et al. 2008; Panyard et al. 2022). Measurement of ptmAAs, on the other hand, which has only recently become more common in targeted metabolomic analysis, offers a clearer picture of the contributions from protein catabolism versus *de novo* synthesis (Darst et al. 2019; Mardinoglu et al. 2015). Here we found many ptmAAs associated with age, indicating that protein catabolism is an important aspect of age‐related metabolome variation. Further support for this hypothesis was provided by the fact that the concentration of ptmAAs within the plasma of dogs was associated with BUN and creatinine (Figure 3b). Together, this evidence points to protein catabolism as a physiological nexus for age‐associated variation in plasma metabolites among companion dogs.

We investigated several potential physiological influences on the abundance of ptmAAs in plasma, looking for those that might explain the age association. Diet data came from owner survey responses, and the most comprehensive variables that we had for this analysis in the current survey instrument was primary diet category. While the survey instrument includes more detailed questions, the response rates at that level of detail were too low to incorporate into this analysis. With the resolution available, we found evidence that diet influences the abundance of only one ptmAA, S‐methylcysteine, which was not age associated.

For there to be more ptmAAs in the plasma of old versus young dogs, the rate of their addition to the plasma must be greater than their rate of removal or metabolism. Studies of digestive enzymes and digestibility in young and old dogs of several breeds, either in a > 100‐dog cohort or in a controlled setting, indicate that older dogs more readily digest protein (Buddington et al. 2003; Weber et al. 2003). Thus, the elevated plasma ptmAAs we found in older dogs may be due in part to increased generation from dietary protein, all else being equal. In addition, we found that a lower rate of removal by glomerular filtration in the kidney in older dogs could explain their elevated ptmAAs. Glomerular filtration rate is typically estimated as a function of the inverse of serum creatinine. While we do not estimate the GFR in this study, we found that approximately 50% of the effect of age on those ptmAAs that were higher in older dogs can be accounted for by variation in creatinine among the dogs (Figure 4b). Similarly, BUN, a complementary indicator of protein metabolism and kidney function, mediates additional ptmAAs (Figure 4c). Creatinine and BUN positively covary with the abundance of ptmAAs generally (Figure 4a), suggesting that high creatinine or BUN, an indication of low GFR, leads to higher ptmAAs. Therefore, the elevated ptmAAs in the plasma of older dogs appears to be due to lower rates of removal by glomerular filtration.

Another indicator of kidney function, uSG (McGlynn et al. 2023), associates with the ptmAA hydroxyproline. Urine specific gravity is a direct measure of the amount of solute removed to the urine and is considered a measure of efficiency in urine concentration. Hydroxyproline can be metabolized into toxic byproducts, and so we would assume that the removal of hydroxyproline to the urine would be at its most efficient at high uSG (Belostotsky and Frishberg 2022). The positive association between uSG and plasma hydroxyproline is therefore paradoxical. However, it is possible that uSG rises and falls directly or otherwise in response to hydroxyproline. Additionally, while maximal uSG is an indicator of renal function, uSG also varies in response to water consumption throughout the day and can also be affected by endocrine disease. Thus, at any given moment, it may not be possible to determine whether a dog's uSG represents its maximal renal concentrating ability, and this may confound the analysis.

Hydroxyproline is a major constituent of collagen and is reasoned to indicate tissue degradation, including muscle wasting, liver injury, and fibrosis, or may reflect the level of dietary animal protein (Keiser et al. 1963). We failed to find an association between hydroxyproline and the primary diet component. An alternative explanation is that its age‐related decline somehow reflects sarcopenia in older dogs. Sarcopenia, the degradation of endogenous muscle tissue with age, is a hallmark of aging in animals (Attaix et al. 2005; Fielding et al. 2011; Saini et al. 2009). Several studies have attempted to characterize serum or plasma metabolites associated with sarcopenia in humans. However, a consensus on plasma biomarkers of sarcopenia has not been reached, including an inconsistent association between plasma hydroxyproline and age‐related muscle loss (Kameda et al. 2020, 2021; Pan et al. 2021). Here, plasma hydroxyproline levels decline in older dogs, which is inconsistent with elevated levels of muscle and liver cell degradation as dogs age.

## 4 Conclusion

The DAP is designed, in part, to develop a companion dog model of aging, which could provide major insights into healthy aging in one of the most variable species of mammal in terms of longevity, behavior, morphology, and pathophysiology (Creevy et al. 2022). We used the age‐associated plasma metabolome as a window into the physiological processes that vary with age in dogs and found that protein catabolism might provide insight into aging. The results presented here come with the important caveat that they represent patterns in a cross‐sectional cohort. As the Dog Aging Project progresses, it will be important to examine longitudinal patterns, asking how metabolites change with age within individual animals as they age. In fact, given the demographic bias that may exist among the baseline Precision Cohort, as with cross‐sectional studies of humans, we may be observing what healthy aging looks like among the older subjects in this study. Having identified age‐associated change in the aqueous metabolome, we have considerable leverage to detect environmental and genetic factors that influence the pace of aging in the longitudinal phases of the DAP, and to identify physiological processes that may respond to aging interventions and their effect on longevity and geriatric health.

## 5 Materials and Methods

The Dog Aging Project (DAP) is a long‐term longitudinal study of companion dogs in the United States. The project is designed to identify the genetic and environmental factors that influence age‐related morbidity and mortality, and the mechanisms by which they do so. Dogs in DAP were recruited with the goal of retaining them for their lifetime. For the Precision Cohort dogs, beginning with the first year of enrollment and at one‐year intervals thereafter, owners bring their dog to their primary care veterinary clinic for biospecimen collection. During this visit, clinical data were recorded, including the dog's age, weight, sex, and sterilization status, as well as the duration of fasting prior to blood collection (Prescott et al. 2025). Additionally, a veterinarian or veterinary technician collects hair, urine, a fecal sample, and blood samples, the last of which were separated into whole blood, plasma, serum, and peripheral blood mononuclear cells (Prescott et al. 2025). One aliquot of plasma was used for targeted aqueous LC–MS metabolomics.

### 5.1 Blood Sampling, Plasma Extraction and Metabolite Extraction

For full details on the design and execution of dog owner contact and sampling in the Precision Cohort, see Prescott et al. (2025). Briefly, blood samples in EDTA tubes, from either one draw of 20 mL for dogs > 8 kg, or two draws of 12 mL each, six weeks apart, for dogs ≤ 8 kg, were shipped to the Texas Veterinary Medical Diagnostic Laboratory. Along with sample appearance and other qualitative checks, the travel time and arrival temperature were recorded. For the samples measured here, the median travel time was 26.3 h (range from 14.2 to 168.7 h). The median arrival temperature was 18.8°C (ranged from 1.8°C to 28.7°C). Plasma was extracted and transferred to 250 μL aliquots in cryovials at the DAP Central Lab at Texas A&M University and stored frozen at −80°C until shipment to the University of Washington (UW). At UW, plasma samples were checked for hemolysis based on the Center for Disease Control and Prevention Hemolysis Reference Palette (CDCHRP, Figure S4). Metabolite extraction was performed at the UW Northwest Metabolomics Research Center (NW‐MRC) in batches of up to 40 samples using a cold‐methanol extraction protocol (Prescott et al. 2025) and stored at −80°C.

Prior to LC–MS, samples were reconstituted in 500 μL HILIC solvent containing ^13^C_2_‐tyrosine, ^13^C_3_‐lactate and 10 mM ammonium acetate in 5% methanol and 0.3% acetic acid. To track secular trends in LC–MS detection that occur during the run, multiple replicates of two different control samples were included in each LC–MS experiment. These include a reference dog plasma sample from the Cornell Veterinary Biobank, referred to as QC(S), and an NW‐MRC human plasma control sample. Each of these was run first, then again, interspersed across the experiment, between every ten DAP samples, and at the end of the run. LC–MS peaks were integrated to give metabolite count data, which were sent from the NW‐MRC back to the Promislow Lab. Raw LC peak and MS spectral data are stored on servers at the NW‐MRC.

The chromatography separations were performed on a duplex‐LC system composed of two Shimadzu UPLC pumps (Shimadzu Corp., Columbia, MD), an Agilent 1290 temperature‐controlled column compartment (Agilent Technologies, Santa Clara, CA), and a CTC Analytics PAL HTC‐xt temperature‐controlled auto‐sampler (LEAP Technologies, Morrisville, NC). The LC modules were controlled by Analyst 1.7.2 software (AB Sciex, Toronto, ON, Canada). Each sample was injected twice, 10 μL for analysis using negative (NEG) ionization mode and 5 μL for analysis using positive (POS) ionization mode. Both chromatographic separations were performed in HILIC mode on two XBridge BEH Amide columns (150 × 2.1 mm, 2.5 μm particle size, Waters Corporation, Milford, MA, Part No. 186009930) connected in parallel. While one column was performing the separation, the other column was reconditioned in preparation for the next injection. The flow rate was 0.300 mL/min, the auto‐sampler temperature was kept at 4°C, the column compartment was set at 45°C, and the total separation time for both ionization modes was 18 min (total analysis time per sample was 36 min). The mobile phase in POS mode was composed of Solvent A (10 mM ammonium acetate in 95% H_2_O/3% acetonitrile/2% methanol + 0.2% acetic acid) and Solvent B (10 mM ammonium acetate in 93% acetonitrile/5% H_2_O/2% methanol + 0.2% acetic acid). The gradient conditions for POS mode separation are shown in Table S2.

### 5.2 Data Transformation and Technical Covariates

The LC–MS data were composed of peak intensity values for 361 metabolites among a total of 1346 samples. This includes 920 baseline Precision Cohort samples, with the remaining samples belonging to other DAP cohorts. These data were collected over the course of five LC–MS runs. In each run, between 73 and 600 samples were run in the same order in which they were processed during metabolite extraction. This design maximizes the statistical power to detect and remove batch and LC–MS run‐order effects. We normalized and pre‐processed all LC–MS data together. We removed 228 metabolites across the study due to missingness in > 10% of all samples. The level of hemolysis was associated with the abundance of some metabolites, and 72 samples were removed due to hemolysis exceeding 500 mg/dL (CDCHRP score = 4, Figure S4). The remaining data were log_e_‐transformed and mean‐centered by sample to account for sample‐to‐sample variation in metabolite abundance.

Both metabolite extraction (batch) and LC–MS runs generate secular variation in metabolite data. Such variation could be due to undefined chemical reactivity, sample matrix effects, ion suppression (interference), etc., and thus differs by metabolite. In these cases, the LC–MS peak corresponding to an affected metabolite drifts with the order in which a sample was processed within a batch. There is also the potential for peak area to vary with the order of samples within an LC–MS run. To correct both for main effects of batch, and for run‐order effects, we take the residuals (e) of the regression model:

(1) metabolite~batch×runorder+e

This results in peak areas that are both normalized and that correct for the effect of run order. To correct for variation in dynamic range among metabolites within experiments, we scaled each metabolite to unit variance by batch. After these procedures, there were an average of 6 missing values per metabolite, with at most 125 missing (9.8%). These missing values were then imputed by 10‐nearest neighbor imputation. Effects of the remaining technical covariates, travel time, hemolysis, and arrival temperature on metabolite abundance were corrected for by linear regression. The data processing and normalization resulted in 133 normalized metabolites measured in 865 dogs in the baseline Precision Cohort.

### 5.3 Breed Ancestry and Genetic Relatedness

Each dog of the Precision Cohort has had low‐pass whole genome sequencing (Sexton et al. 2025). Briefly, reads were aligned to the CanFam3.1 reference genome assembly (NCBI accession GCF_000000145.2) and imputation using a panel of reference haplotypes including > 34 M SNPs and > 11 M indels shared by 109 modern dog breeds, 3 village dog populations, and North American and European wolf populations (Sexton et al. 2025). SNPs with a minor allele frequency greater than 1% and genotype call rates greater than 95% were retained. Here we used the genetic data in two ways—first, to determine breed by genetic ancestry and evaluate the effect of such breed information on metabolome profiles, and second, to control for relatedness among all dogs regardless of breed. For breed ancestry, we estimated the proportion of genetic ancestry in each dog genome using publicly available genotype data from 109 modern breeds, village dog populations from three regions, and two wolf populations. Ancestry proportions for each dog were then estimated using ADMIXTURE on genotype data from 115,427 biallelic SNPs. Where applicable here, purebred was defined as any dog with ≥ 85% ancestry assigned to a single breed (Morrill et al. 2022). To estimate relatedness, the variance‐standardized GRM was calculated with autosomal markers in PLINK2 using the default settings in the make‐rel function (Chang et al. 2015).

### 5.4 Variable Selection and Normalization

Of the data collected on Precision Cohort dogs, age and weight were square root transformed, and sex (male and female) and sterilization status (intact or sterile) were coded as factors (i.e., 0 and 1). The duration of fasting prior to blood collection was rounded to the nearest hour. Based on the American Animal Hospital Association's Canine Life Stage Guidelines, Precision Cohort dogs were classified into one of five age categories: puppy (< 1 year), adolescent (1–3 years), young adult (3–7 years), mature adult (7–11 years), and senior (11+ years) (Prescott et al. 2025).

Complete Blood Count and blood chemistry (CBC/Chem) data were acquired at the Texas Veterinary Medical Diagnostic Laboratory from samples in the DAP biospecimen kits. CBC measures were taken from blood samples in EDTA tubes on an Advia 120 Hematology System (Siemens Medical Solutions, Malvern, PA) from 784 of the Precision Cohort dogs that had metabolome profiles. Parallel blood chemistry profiles were performed with serum extracted from an accompanying additive‐free tube and run on a DxC700AU Chemistry Analyzer (Beckman Coulter, Brea, CA). Together the raw data consist of 45 CBC and 42 chemistry measures. The 36 dogs without CBC/Chem data were removed from further analysis. Of the 45 CBC traits, we removed five invariant measures, 36 that had > 10 missing values, and any that were relative measures when an absolute measure was available. Among the remaining 39 variables, ten of the numerical CBC variables were non‐normal, including counts of band cells, neutrophils, lymphocytes, monocytes, eosinophils, basophils, and reticulocytes, red cell distribution width, mean platelet volume, and plateletcrit (Shapiro–Wilk statistic < 0.96), and were log_e_‐transformed. There was an average of one missing entry among each of the variables at this point (maximum missingness was five). All missing data were imputed by 10‐nearest neighbor mean imputation, which gave 38 complete and normalized CBC/Chem variables in 784 dogs (Table S3). The effects of blood sample travel time and arrival temperature on each CBC trait were removed by linear regression. The adjusted 17 CBC variables were used as covariates in mixed models.

Urinalysis was performed with approximately 3 mL of urine on 738 of these 784 dogs. The uSG was calculated by refractometer; chemical analysis was performed with Multistix 10 SG Urine Test Strips (Siemens Medical Solutions, Malvern, PA); and microscopy was performed manually (Prescott et al., in prep). After removing two invariant urinalysis variables, there were two numeric variables: uSG and pH, and 12 categorical variables. Eight of the categorical variables: protein, white blood cells, red blood cells, squamous cells, urothelial cells, bilirubin, fat, and blood, were ordered semi‐quantitatively. For example, urothelial and other cell counts were coded “None Observed” < “Rare” < “0–3” < “3–6” < “6–10” < “10–20” < “20–40” < “Too numerous to count”. The remaining categorical variables, including urine color, transparency, crystals, and casts, were not evaluated. This gave 10 clinical urinalysis variables (Table S3). As covariates, the ordered categories were converted to integers and, along with the continuous numeric variables, were mean‐centered and scaled to unit variance prior to model fitting with each as a fixed effect, using the mixed model described below (Equation 2).

### 5.5 Principal Components Analysis

Principal component analysis was performed on normalized metabolome data from 784 dogs for which we also had CBC data, with additional scaling by metabolite. We used the Tracy‐Widom test in the *AssocTest* package to identify the first 22 PCs that describe significantly non‐random variation (*α* = 0.05). Type III ANCOVA was used to estimate the variance of each of the first 22 PCs that could be explained by the effects of each covariate.

### 5.6 Linear Mixed Model

The fixed effects (β) of X, the design matrix of the covariates: age, weight, sex, sterilization status, the duration of fasting prior to blood collection, the 17 CBC traits, and the interactions between age and weight, and sex and sterilization status, were fit simultaneously on each metabolite (y), along with the random effects $\hat{u}$ (best linear unbiased predictions, BLUPs) of the covariance in the GRM.

(2) $$y=\mathrm{X\beta}+Z\hat{u}+e$$

The mixed model was fit by maximum likelihood in the *EMMREML* package. We tested for significance (β > 0) of fixed effects within the emmreml function, and the *p* values were corrected for multiple comparisons by the false discovery rate method (FDR, Benjamini and Hochberg 1995) When comparing models with and without the GRM, we fit an identity matrix (diagonals = 1, off‐diagonals = 0) in place of the GRM.

### 5.7 Adjustment for Fixed and Random Effects

When assessing the correlation among metabolites, creatinine and BUN, we removed the effects of the fixed covariates from the metabolite values (y) by subtracting both fixed effects from Equation 2 (the best linear unbiased estimators, BLUEs), and random effects (BLUPs) from Equation 2 using Equation 3:

(3) $$y^{\prime }=y-\text{BLUEs}-\text{BLUPs}$$

where BLUEs were derived by multiplying the design matrix (X) by the matrix of fixed effects (β), which, when subtracted from y, give the fully adjusted values ($y^{\prime }$).

### 5.8 Mediation Analysis

We performed mediation analysis with the *mediation* R package (Tingley et al. 2014), testing for the causal mediation effect (γ) of a mediator (M) on the effect of age (β) on a metabolite. Mediation was estimated in linear models with the same fixed‐effects covariates (X) used in the mixed model (Equation 2), without the random effect of the GRM (Equation 4):

(4) $$\text{metabolite}=\beta \;\mathrm{age}+\gamma \left(M\right)+X+e$$

The causal mediation effect (Figure S5) was estimated and tested for γ > 0 using up to 10^6^ bootstrap randomizations of age. The proportion mediated is given by dividing γ by the effect of age on a metabolite without the mediator (Figure S5). We tested the sensitivity of γ to unmeasured confounding among the predictors by sensitivity analysis, where correlation (ρ) between the residual effects of the mediator and outcome variables was artificially introduced to estimate the ρ at which γ = 0 (Imai et al. 2010). None of the mediation models with γ > 0 at FDR ≤ 5% were sensitive.

## Author Contributions

Conception and design were by K.E.C., D.E.L.P., N.S.‐M., A.A., D.R., M.D.D., and B.R.H. Data collection, analysis, consultation, and interpretation were by B.R.H., M.P.‐A., A.M., D.D., M.K., B.L.M., B.M.M., Y.M.A., E.M., D.R., K.E.C., A.A., E.B., N.S.‐M., and D.E.L.P. Writing and revision were done by all authors.

## Conflicts of Interest

D.E.L.P. is on the board of Wndrhlth Club Inc.

## Supporting information


**Figure S1:** The multivariate dog plasma metabolome (a) Analysis of covariance (ANCOVA) sum of squares (SS) among the covariates (ANCOVA term) within each of the first 22 principal components (PC) of the plasma metabolome. The residual SS not accounted for by the terms is shown in gray. In (a) the SS from the 17 CBC traits are combined and indicated by the term “CBC” (pink). (b) The SS for each of the 17 CBC traits across the first 22 PCs of the metabolome.
**Figure S2:** Breed has complex effects on the metabolome, partly accounted for by relatedness (a) The 5 principal components (PCs) with effects of the 8 common breeds (ANCOVA *p* < 0.05) plotted by breed, including a single category for all other dogs (remaining dogs). Within each plot, breeds are ordered by the mean weight of the dogs in each breed (*n* = 8–44 cohort dogs per breed). (b) The average variance among the 5 PCs in (a) that is accounted for by the fixed effects indicated on the *x*‐axis (Methods) in models that either include a random effect of relatedness (by including the genetic relatedness matrix, +GMR), or not (naïve, Methods). The percent reduction in average variance when the GRM is included is indicated for the two most affected terms (breed and sterilization).
**Figure S3:** Primary diet composition does not explain variation in post‐translationally modified amino acids in plasma. (a) The distribution of the primary diet type among owner survey responses of 761 dogs, with counts plotted on a log scale. Diet components were divided into those commercially sourced (Commercial), home prepared (Home), or of some other type (Other). Other reflects diet components that do not fit one of the seven categories or where diet was not consistent, non‐responses were omitted. (b) A heatmap showing the effect of diet type on each metabolite (𝛽_diet_) in comparison to dry kibble in a mixed model to control for age, weight, and other covariates (Methods). Alongside the metabolite values among the reference diet, only metabolites and diets that had at least one effect are shown (𝛽_diet_ ≠ 0, FDR < 0.05, asterisks). (c) S‐methylcysteine was the only ptmAA to associate with any primary diet component (FDR < 5%, asterisks).
**Figure S4:** Hemolysis reference palette. The colorimetric reference palette used by the Dog Aging Project (DAP) to assess plasma sample hemolysis. Plasma samples in polypropylene microfuge tubes were compared to the Center for Disease Control and Prevention (CDC) palette and given a score from 1 to 4. Samples with a score of 4 were not analyzed.
**Figure S5:** Path representation of causal mediation models. A path representation of a causal mediation model using, as an example, the hypothetical effect of age on hydroxyproline, with urine specific gravity as a potential mediator. The mediation model (path a‐b), was compared to a model without urine specific gravity (path c). The total effect was path c, without considering path a‐b. To detect causal mediation, after satisfying the assumption that path a was significant, the direct effect (c|b) of urine specific gravity was the effect of path c given path b. The mediation effect was the total effect minus the direct effect, and its significance (γ > 0) was tested by bootstrap resampling age in the model (Methods). The proportion mediated was γ divided by the total effect.

**Table S1:** Age‐associations among 133 plasma metabolites in the plasma of the precision cohort.

**Table S2:** Liquid chromatography gradient conditions.

**Table S3:** Summary of complete blood count, serum chemistry and urinalysis variables.

## Data Availability

Dog Aging Project data are available on the TERRA platform (https://app.terra.bio/). Code will be made available on GitHub upon publication.

## Appendix A

### Dog Aging Project Consortium

Joshua M. Akey^a^ | Rozalyn M. Anderson^b^ | Elhanan Borenstein^c^ | Marta G. Castelhano^d^ | Amanda E. Coleman^e^ | Kate E. Creevy^f^ | Matthew D. Dunbar^g^ | Virginia R. Fajt^h^ | Jessica M. Hoffman^i^ | Erica C Jonlin^j^ | Matt Kaeberlein^k^ | Elinor K. Karlsson^l^ | Kathleen F. Kerr^m^ | Jing Ma^n^ | Evan L. MacLean^o^ | Stephanie McGrath^p^ | Natasha J Olby^q^ | Daniel E.L. Promislow^r^ | May J Reed^s^ | Audrey Ruple^t^ | Stephen M. Schwartz^u^ | Sandi Shrager^v^ | Noah Snyder‐Mackler^w^ | M. Katherine Tolbert^f^

^a^Lewis‐Sigler Institute for Integrative Genomics, Princeton University, Princeton, NJ, USA | ^b^Department of Medicine, University of Wisconsin ‐ Madison, Madison, WI, USA | ^c^Department of Clinical Microbiology and Immunology, Gray Faculty of Medical and Health Sciences, Tel Aviv University, Tel Aviv, Israel | ^d^Cornell Veterinary Biobank, College of Veterinary Medicine, Cornell University, Ithaca, NY, USA | ^e^Department of Small Animal Medicine and Surgery, College of Veterinary Medicine, University of Georgia, Athens, GA, USA | ^f^Department of Small Animal Clinical Sciences, Texas A&M University School of Veterinary Medicine & Biomedical Sciences, College Station, TX, USA | ^g^Center for Studies in Demography and Ecology, University of Washington, Seattle, WA, USA | ^h^Department of Veterinary Physiology and Pharmacology, Texas A&M University School of Veterinary Medicine & Biomedical Sciences, College Station, TX, USA | ^i^Department of Biological Sciences, Augusta University, Augusta, GA, USA | ^j^Department of Laboratory Medicine and Pathology, University of Washington School of Medicine, Seattle, WA, USA | ^k^Optispan, Inc. Seattle, WA, USA | ^l^Bioinformatics and Integrative Biology, University of Massachusetts Chan Medical School, Worcester, MA, USA | ^m^Department of Biostatistics, University of Washington, Seattle, WA, USA | ^n^Division of Public Health Sciences, Fred Hutchinson Cancer Research Center, Seattle, WA, USA | ^o^College of Veterinary Medicine, University of Arizona, Tucson, AZ, USA | ^p^Department of Clinical Sciences, College of Veterinary Medicine and Biomedical Sciences, Colorado State University, Ft. Collins, CO, USA | ^q^Department of Clinical Sciences, College of Veterinary Medicine, North Carolina State University, Raleigh, NC, USA | ^r^Jean Mayer USDA Human Nutrition Research Center on Aging at Tufts University, Boston, MA, USA | ^s^Department of Medicine, Division of Gerontology and Geriatric Medicine, University of Washington School of Medicine, Seattle, WA, USA | ^t^Department of Population Health Sciences, Virginia‐Maryland College of Veterinary Medicine, Virginia Tech, Blacksburg, VA, USA | ^u^Epidemiology Program, Fred Hutchinson Cancer Center, Seattle, WA, USA | ^v^Collaborative Health Studies Coordinating Center, Department of Biostatistics, University of Washington, Seattle, WA, USA | ^w^School of Life Sciences, Arizona State University, Tempe, AZ, USA | ^f^Department of Small Animal Clinical Sciences, Texas A&M University School of Veterinary Medicine & Biomedical Sciences, College Station, TX, USA.
