# Supplementary material for: Protein Catabolites as Blood‐Based Biomarkers of Aging Physiology: Findings From the Dog Aging Project
Source: Aging Cell. 2025 Oct 22;24(11):e70226. doi: 10.1111/acel.70226 (PMC12611326; doi:10.1111/acel.70226)
Supplement: Supplementary file 1 — Figure S1: The multivariate dog plasma metabolome (a) Analysis of covariance (ANCOVA) sum of squares (SS) among the covariates (ANCOVA term) within each of the first 22 principal components (PC) of the plasma metabolome. The residual SS not accounted for by the terms is shown in gray. In (a) the SS from the 17 CBC traits are combined and indicated by the term “CBC” (pink). (b) The SS for each of the 17 CBC traits across the first 22 PCs of the metabolome. Figure S2: Breed has complex effects on the metabolome, partly accounted for by relatedness (a) The 5 principal components (PCs) with effects of the 8 common breeds (ANCOVA p < 0.05) plotted by breed, including a single category for all other dogs (remaining dogs). Within each plot, breeds are ordered by the mean weight of the dogs in each breed (n = 8–44 cohort dogs per breed). (b) The average variance among the 5 PCs in (a) that is accounted for by the fixed effects indicated on the x‐axis (Methods) in models that either include a random effect of relatedness (by including the genetic relatedness matrix, +GMR), or not (naïve, Methods). The percent reduction in average variance when the GRM is included is indicated for the two most affected terms (breed and sterilization). Figure S3: Primary diet composition does not explain variation in post‐translationally modified amino acids in plasma. (a) The distribution of the primary diet type among owner survey responses of 761 dogs, with counts plotted on a log scale. Diet components were divided into those commercially sourced (Commercial), home prepared (Home), or of some other type (Other). Other reflects diet components that do not fit one of the seven categories or where diet was not consistent, non‐responses were omitted. (b) A heatmap showing the effect of diet type on each metabolite (𝛽diet) in comparison to dry kibble in a mixed model to control for age, weight, and other covariates (Methods). Alongside the metabolite values among the reference diet, only metabo [file ACEL-24-e70226-s001.pptx]

## Slide 1
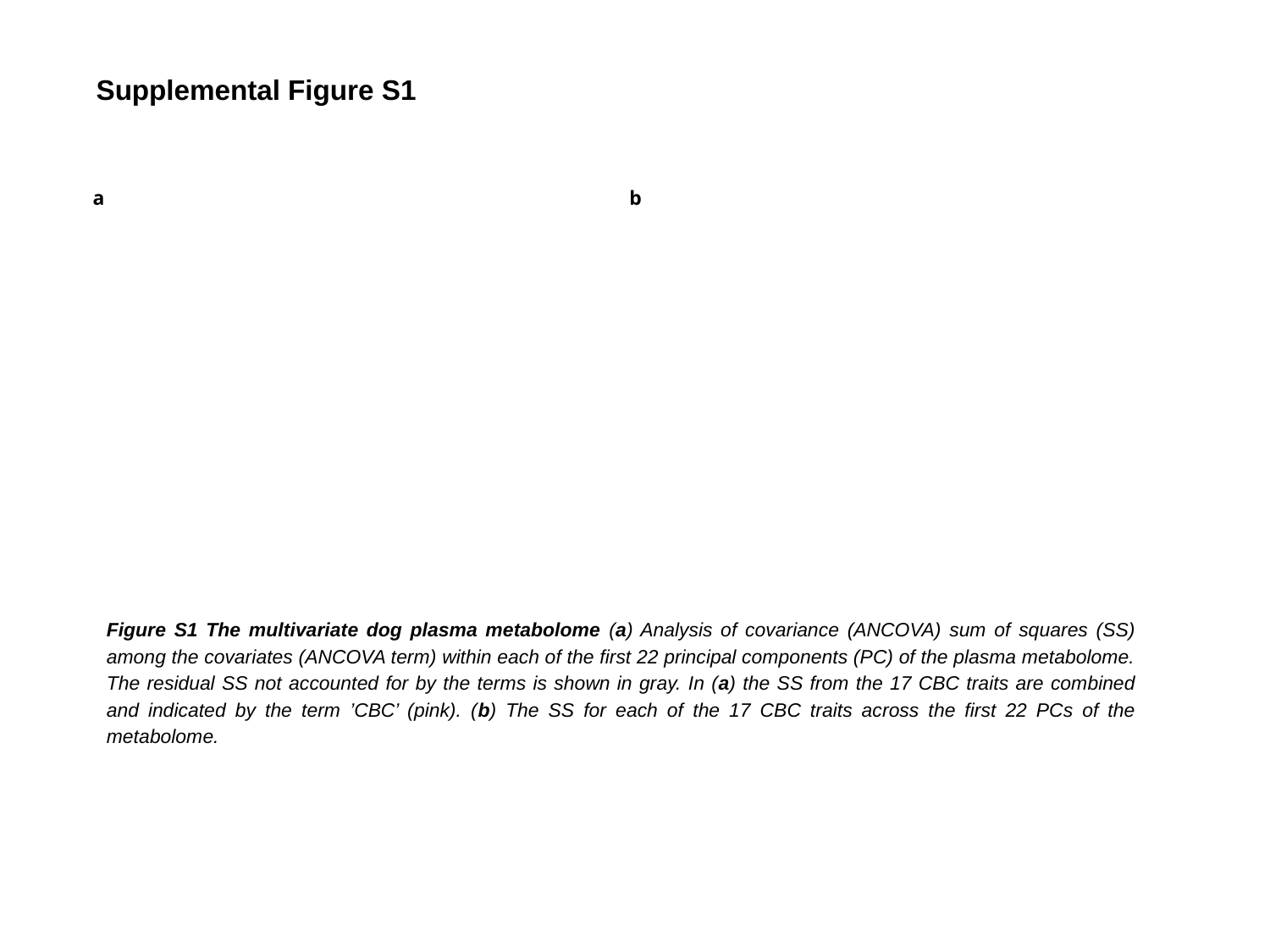

Supplemental Figure S1
a
b
Figure S1 The multivariate dog plasma metabolome (a) Analysis of covariance (ANCOVA) sum of squares (SS) among the covariates (ANCOVA term) within each of the first 22 principal components (PC) of the plasma metabolome. The residual SS not accounted for by the terms is shown in gray. In (a) the SS from the 17 CBC traits are combined and indicated by the term ’CBC’ (pink). (b) The SS for each of the 17 CBC traits across the first 22 PCs of the metabolome.

## Slide 2
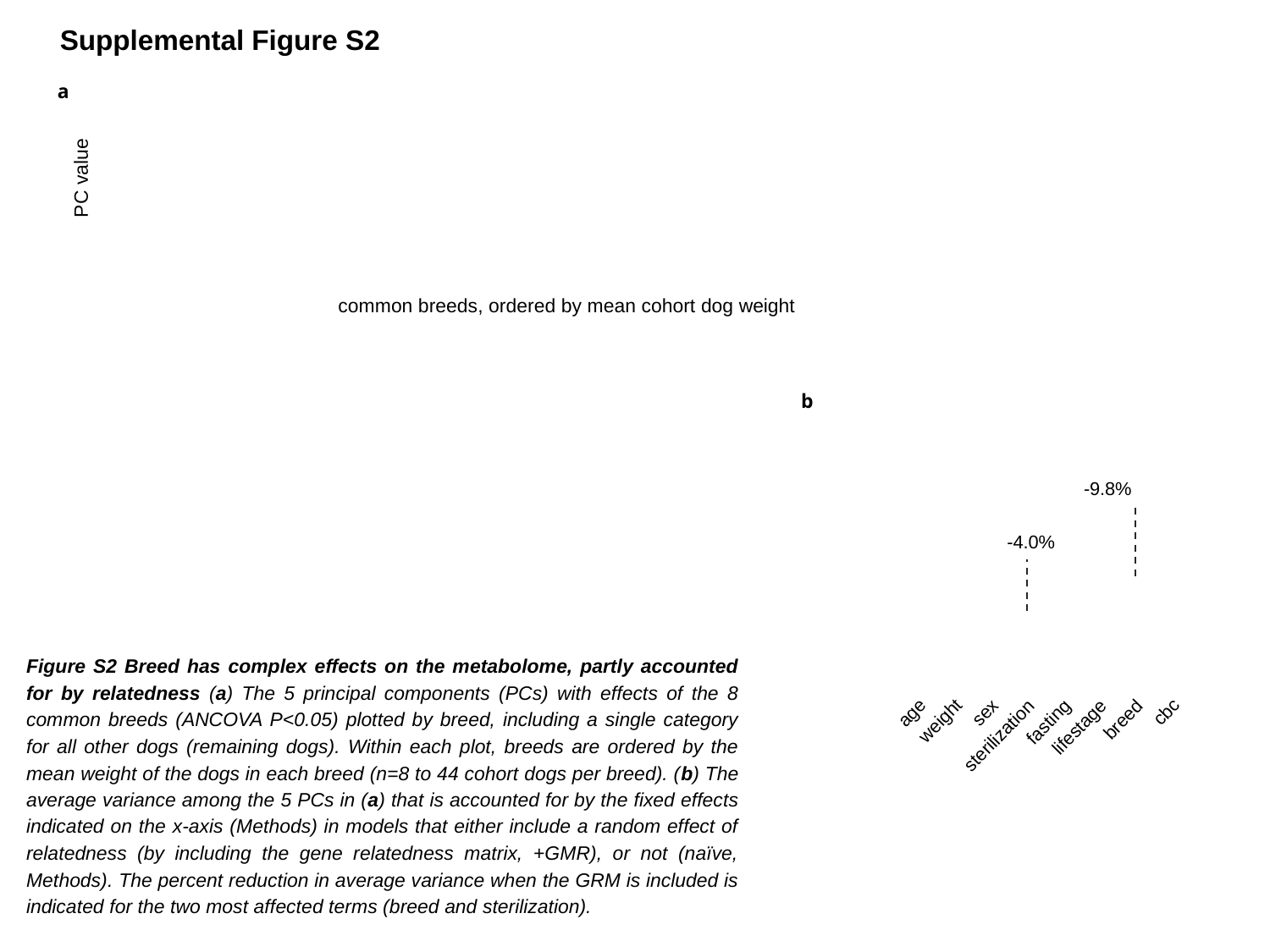

Supplemental Figure S2
a
PC value
common breeds, ordered by mean cohort dog weight
b
-9.8%
-4.0%
Figure S2 Breed has complex effects on the metabolome, partly accounted for by relatedness (a) The 5 principal components (PCs) with effects of the 8 common breeds (ANCOVA P<0.05) plotted by breed, including a single category for all other dogs (remaining dogs). Within each plot, breeds are ordered by the mean weight of the dogs in each breed (n=8 to 44 cohort dogs per breed). (b) The average variance among the 5 PCs in (a) that is accounted for by the fixed effects indicated on the x-axis (Methods) in models that either include a random effect of relatedness (by including the gene relatedness matrix, +GMR), or not (naïve, Methods). The percent reduction in average variance when the GRM is included is indicated for the two most affected terms (breed and sterilization).
cbc
sex
age
breed
weight
fasting
lifestage
sterilization

## Slide 3
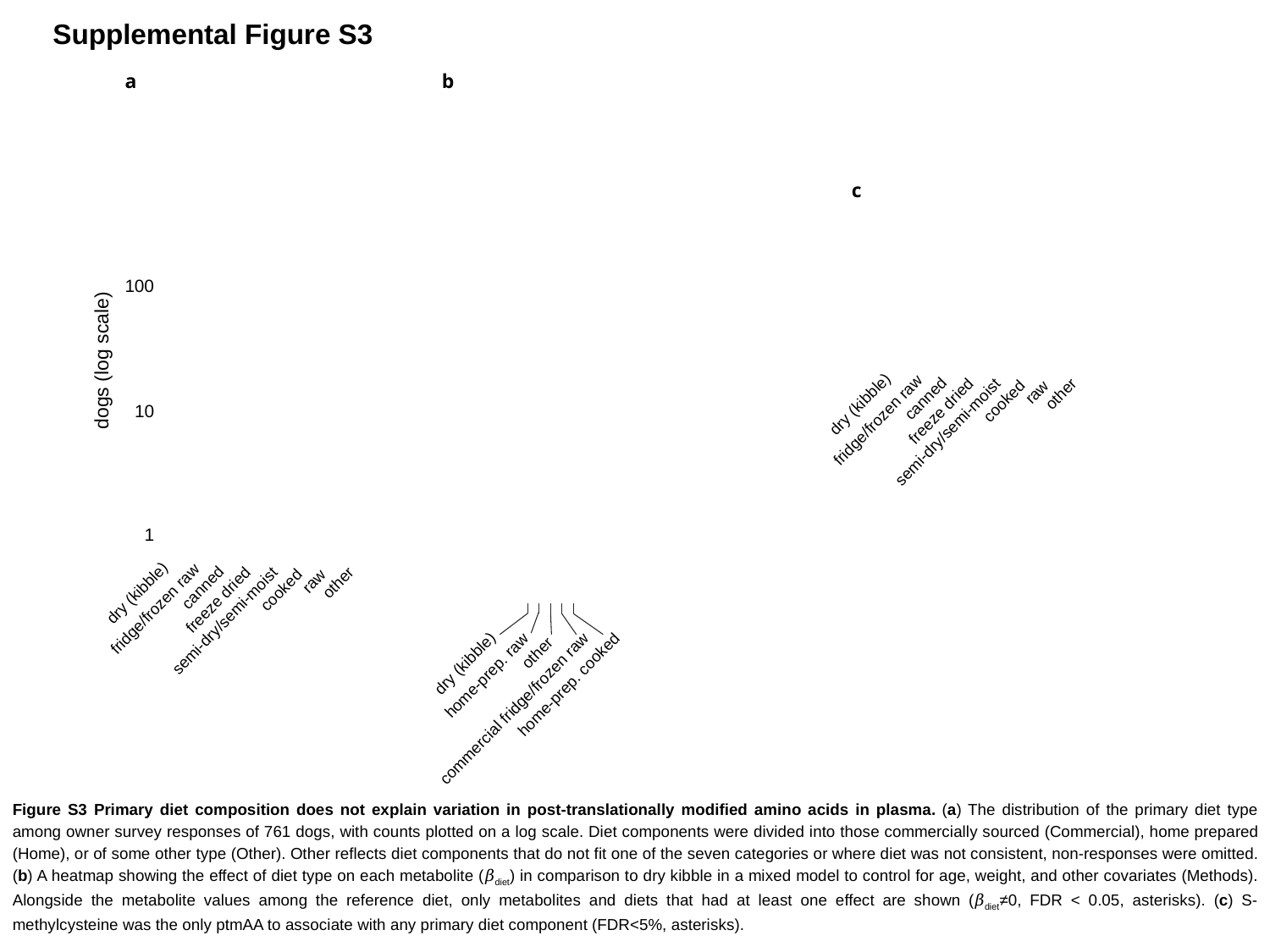

Supplemental Figure S3
a
b
other
dry (kibble)
home-prep. cooked
commercial fridge/frozen raw
c
100
dogs (log scale)
raw
other
canned
cooked
dry (kibble)
10
freeze dried
fridge/frozen raw
semi-dry/semi-moist
1
raw
other
canned
cooked
dry (kibble)
freeze dried
fridge/frozen raw
semi-dry/semi-moist
home-prep. raw
Figure S3 Primary diet composition does not explain variation in post-translationally modified amino acids in plasma. (a) The distribution of the primary diet type among owner survey responses of 761 dogs, with counts plotted on a log scale. Diet components were divided into those commercially sourced (Commercial), home prepared (Home), or of some other type (Other). Other reflects diet components that do not fit one of the seven categories or where diet was not consistent, non-responses were omitted. (b) A heatmap showing the effect of diet type on each metabolite (𝛽diet) in comparison to dry kibble in a mixed model to control for age, weight, and other covariates (Methods). Alongside the metabolite values among the reference diet, only metabolites and diets that had at least one effect are shown (𝛽diet≠0, FDR < 0.05, asterisks). (c) S-methylcysteine was the only ptmAA to associate with any primary diet component (FDR<5%, asterisks).

## Slide 4
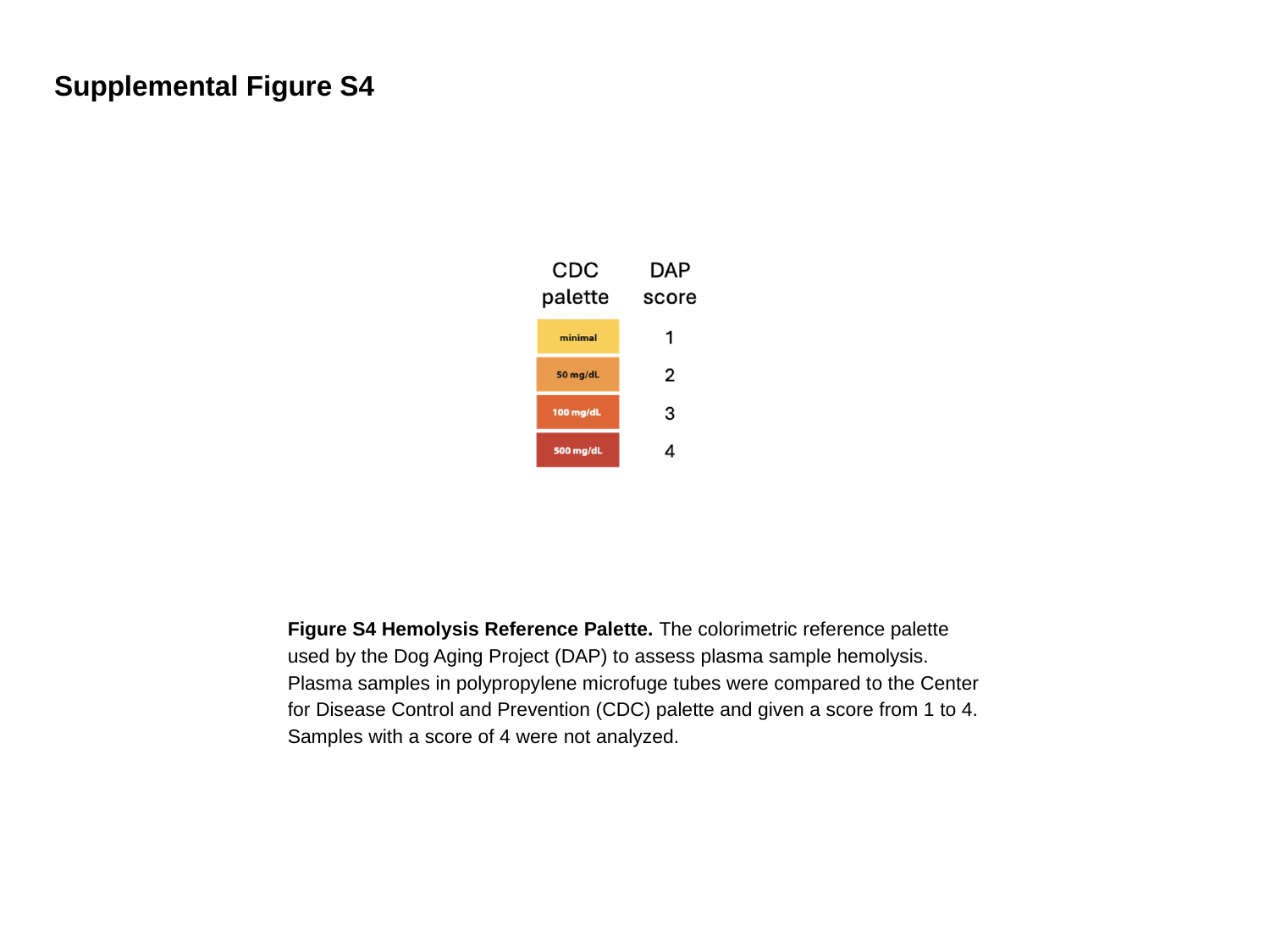

Supplemental Figure S4
Figure S4 Hemolysis Reference Palette. The colorimetric reference palette used by the Dog Aging Project (DAP) to assess plasma sample hemolysis. Plasma samples in polypropylene microfuge tubes were compared to the Center for Disease Control and Prevention (CDC) palette and given a score from 1 to 4. Samples with a score of 4 were not analyzed.

## Slide 5
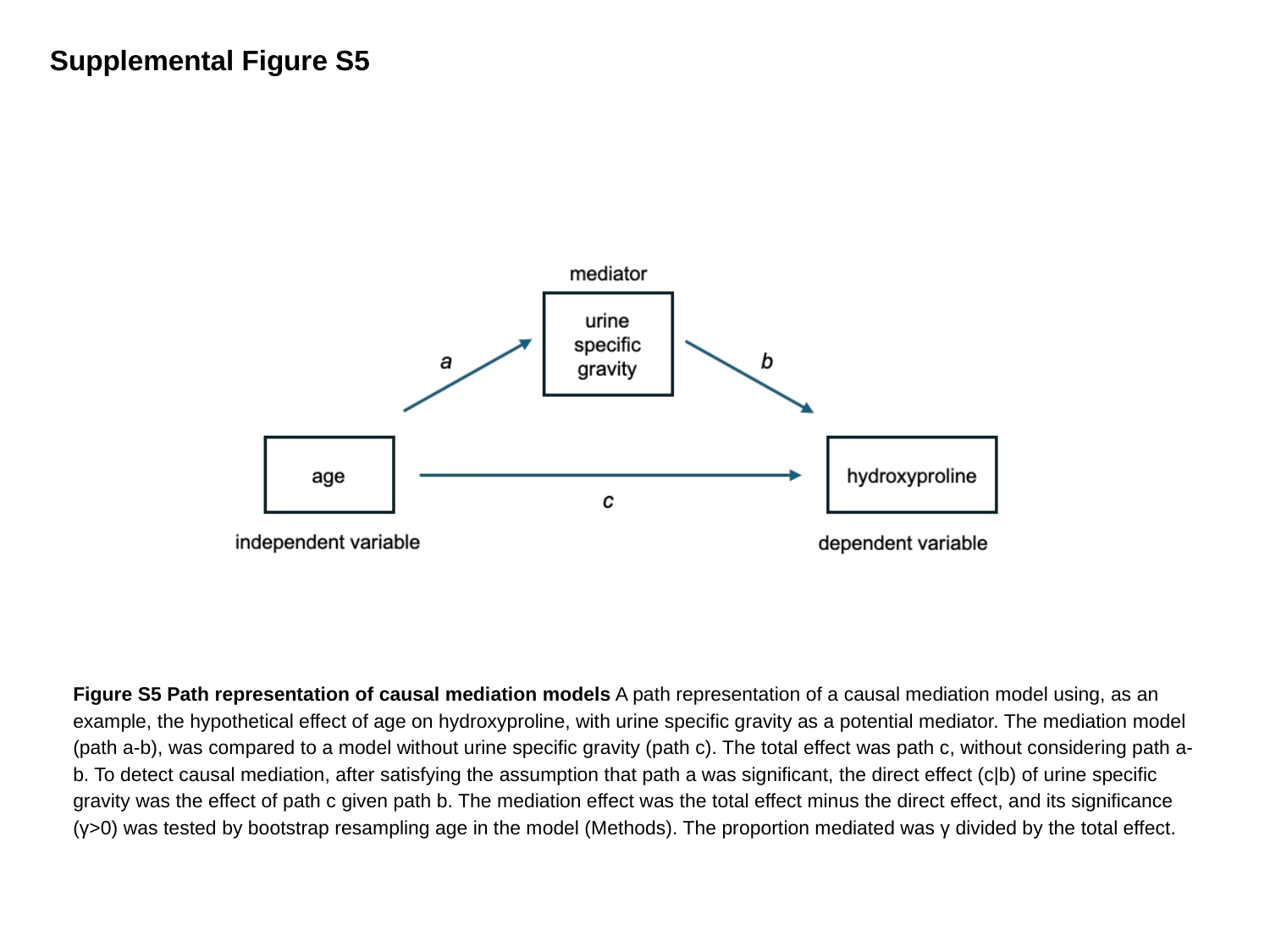

Supplemental Figure S5
Figure S5 Path representation of causal mediation models A path representation of a causal mediation model using, as an example, the hypothetical effect of age on hydroxyproline, with urine specific gravity as a potential mediator. The mediation model (path a-b), was compared to a model without urine specific gravity (path c). The total effect was path c, without considering path a-b. To detect causal mediation, after satisfying the assumption that path a was significant, the direct effect (c|b) of urine specific gravity was the effect of path c given path b. The mediation effect was the total effect minus the direct effect, and its significance (γ>0) was tested by bootstrap resampling age in the model (Methods). The proportion mediated was γ divided by the total effect.
